# Perceptions of female age, health and attractiveness vary with systematic hair manipulations

**DOI:** 10.1111/ics.70028

**Published:** 2025-10-20

**Authors:** Susanne Will, Mandy Beckmann, Kristina Kunstmann, Julia Kerschbaumer, Yu Lum Loh, Samuel Stofel, Paul J. Matts, Todd K. Shackelford, Bernhard Fink

**Affiliations:** ^1^ The Procter & Gamble Company Schwalbach am Taunus Germany; ^2^ The Procter & Gamble Company, The Metropolis Singapore City Singapore; ^3^ The Procter & Gamble Company Cincinnati Ohio USA; ^4^ The Procter & Gamble Company Weybridge UK; ^5^ Department of Psychology Oakland University Rochester Michigan USA; ^6^ Biosocial Science Information Biedermannsdorf Austria; ^7^ Human Evolution and Archaeological Sciences (HEAS) University of Vienna Vienna Austria; ^8^ Department of Evolutionary Anthropology University of Vienna Vienna Austria

**Keywords:** alignment, attractiveness, hair, perception, shine, women

## Abstract

**Objective:**

Research on female facial attractiveness has focused on the effects of face shape and skin condition. Few studies have investigated the impact of hair on assessments of female attractiveness. Research using images of computer‐generated (rendered) hair has demonstrated that subtle variations in hair thickness, density and style affect perceptions of female age, health and attractiveness.

**Method:**

The current study consisted of two experiments in which non‐expert female panellists viewed distinct expressions of specific hair features and judged them for age, health and attractiveness. In Experiment 1, women from three countries (Germany, Spain and USA; *n* = 500 each) judged high‐shine and low‐shine versions of natural Caucasian hair wigs on a female target—photographed from right back (3/4 view)—for age, health and attractiveness. In Experiment 2, professional stylists manipulated shine, alignment and volume of natural Caucasian hair wigs, creating two versions—one high and one low in each feature—for blonde hair and brown hair. A woman with light skin pigmentation wore the wigs and was photographed in three head orientations under controlled lighting conditions. Omnibus pairwise combinations of hair conditions were created and judged by *n* = 2000 US women for age, health and attractiveness.

**Results:**

Experiment 1 showed that, across countries, high‐shine hair was rated more youthful, healthier and attractive than low‐shine hair. Experiment 2 indicated that straight‐aligned hair was perceived as most youthful, healthy and attractive, regardless of hair colour and head orientation. High shine also was preferred, although its impact was weaker than that of hair alignment.

**Conclusion:**

Straight‐aligned hair, together with shine, affects female appearance and this influence is noticeable even with small (mobile phone‐sized) images.

## Graphical abstract

Two experiments were conducted to study how lay individuals evaluate systematic changes in female head hair. The results showed that participants tended to rate high‐shine hair more favourably than lower‐shine hair. When combining shine with alignment and volume, straight‐aligned hair with high shine and low volume was generally perceived as more youthful, healthy and attractive.

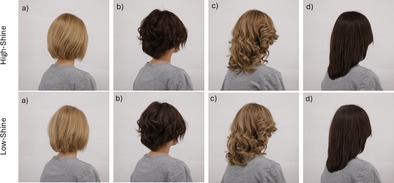

## Résumé

**Objectif:**

Les recherches sur l'attrait du visage féminin se sont surtout concentrées sur les effets de la forme du visage et de l'état de la peau. Peu d'études se sont intéressées à l'impact de la chevelure dans l'évaluation de l'attractivité féminine. Des travaux menés à partir d'images de cheveux générés par ordinateur ont montré que de légères variations d'épaisseur, de densité et de style influencent la perception de l'âge, de la santé et de l'attrait des femmes.

**Méthode:**

La présente étude se compose de deux expériences dans lesquelles des panélistes féminines non expertes ont observé différentes expressions de caractéristiques capillaires précises et les ont évaluées en termes d'âge, de santé et d'attractivité. Dans l'Expérience 1, des femmes de trois pays (Allemagne, Espagne et États‐Unis; *n* = 500 chacun) ont évalué des versions à forte et faible brillance de perruques en cheveux caucasiens naturels portées par une cible féminine, photographiée de dos en trois‐quarts, selon les critères d'âge, de santé et d'attractivité. Dans l'Expérience 2, des coiffeurs professionnels ont manipulé la brillance, la discipline et le volume des perruques en cheveux caucasiens naturels, créant deux versions (une accentuée et une atténuée pour chaque caractéristique) sur cheveux blonds et bruns. Une femme à la peau claire portait ces perruques et a été photographiée dans trois orientations de tête différentes, sous des conditions d'éclairage contrôlées. Des combinaisons par paires complètes des différentes conditions capillaires ont été créées et évaluées par *n* = 2000 femmes américaines selon l'âge, la santé et l'attractivité.

**Résultats:**

L'Expérience 1 a montré que, dans tous les pays, les cheveux très brillants étaient jugés plus jeunes, plus sains et plus attrayants que les cheveux ternes. L'Expérience 2 a indiqué que les cheveux lisses et disciplinés étaient perçus comme plus jeunes, plus sains et plus attrayants, indépendamment de la couleur des cheveux et de l'orientation de la tête. Une forte brillance a également été préférée, bien que son impact ait été moins marqué que celui de la discipline.

**Conclusion:**

Les cheveux lisses et disciplinés, associés à la brillance, influencent l'apparence féminine, et cette influence demeure perceptible même sur de petites images (de la taille d'un écran de téléphone portable).

## INTRODUCTION

Research investigating perceptions of female physical appearance typically has considered either the entire face (e.g., symmetry [1, 2]) or specific facial features (e.g., shape of eyes [3]; mouth/lips [4]) or body features (e.g., curvaceousness [5]). From an evolutionary perspective, evaluating physical appearance is important in social contexts, as it influences initial impressions and trait attributions of unfamiliar individuals [6, 7]. According to evolutionary logic, physical features can serve as ‘honest’ cues to an individual’s mating‐related qualities [8, 9, 10, 11]. Assessments of youth and health strongly correlate with female reproductive potential [12]. The physical appearance of a prospective mate matters for both men and women. However, research indicates a sex difference in many populations, with men placing greater importance than women on the physical attractiveness of a prospective mate [13, 14].

Studies investigating facial appearance [15] digitally manipulated features using image morphing technology [16, 17], exaggerating features hypothesized to vary with facial attributions. Research using geometric morphometric methodology [18] has explored relationships between facial shapes based on facial landmarks and target traits [19]. Other research has investigated the impact of skin condition on facial perception [20]. Across populations, skin surface topography and skin coloration affect assessments of female age, health and attractiveness, with local variation attributable to socio‐ecological conditions [21, 22].

Face research typically removes information about head hair. By imaging participants with a hairband or cap, or digitally removing hair, observers in rating studies do not have access to information about the participants' hair. Head hair may not reliably indicate genetic quality related to mating (but see [23] for evidence of a positive relationship between hair quality/length and sexual activity), as its colour and style can be highly variable and easily altered. However, research using computer‐generated (rendered) hair documented variation in age, health and attractiveness assessments, depending on hair diameter, density and style [24]. Matz and Hinsz [25] found that hair colour and length influence personality perceptions. In addition, women with healthier‐looking hair are judged to have greater reproductive health [26]. Changes in hair appearance due to ageing or poor health can be attributed to factors such as alterations in hair diameter, density and colour (e.g., greying) [27, 28]. These changes may result in less favourable assessments of health and attractiveness [29, 30, 31]. Fink et al. [24] showed that even subtle changes in hair characteristics impact hair perception. These changes depend on hair type (straight vs. wavy) and colour. For example, straight hair, independent of diameter, was perceived as more youthful than wavy hair. Interactions between hair type and colour were more evident in health and attractiveness ratings. Straight brown hair was rated healthiest, whereas ash blonde hair was rated most attractive (see also [32]). Research on the role of hair colour in perceptions of physical appearance has produced mixed results. Some studies (e.g., [33]) have suggested a ‘rare‐colour advantage’, indicating that women with less common hair colours are perceived as more attractive than those with more common hair colours (but see [34]). Other studies reported that women with blonde hair are rated as less physically attractive and more sexually promiscuous than women with medium brown (‘brunette’) hair [35, 36].

Examining an individual variable, such as hair colour, yields limited insights due to the complex and multifaceted nature of hair assessments. This corresponds with evidence indicating that hormonal changes influence hair characteristics [30, 37, 38, 39]. Also, head hair can be altered through dyeing and styling, alongside age‐related changes [24, 40]. Moreover, the evaluation of female physical appearance, encompassing face and hair characteristics, results in interaction effects between these features [41, 42]. Hair shine's impact on female physical appearance has not been studied in vivo, although it has been suggested that shiny, healthy hair is attractive [29, 43]. Research on natural and coloured hair tresses documents the effect of mechanical damage (e.g., via a combing wheel) on visual attention and assessments of hair [44]. People looked longer at undamaged coloured hair and rated it higher in health and attractiveness than damaged hair. The researchers concluded that differences in social perception and visual attention are due to changes in the visual appearance of undamaged and damaged hair. Physical damage affected hair's optical properties, leading to lower shine and these subtle visual cues affected judgements of age, health and attractiveness [44].

The present research extends previous work on the impact of hair shine on perceptions of female hair by systematically manipulating natural hair wigs and recording assessments from non‐expert raters. Light interacting with hair fibres creates complex visual patterns through reflection, absorption, and scattering [45, 46, 47]. The mixed evidence on hair perception from previous research may be due to the choice of stimuli that did not account for the interaction of hair fibres with light, leading to the special visual appearance of human hair [44, 46, 48]. Unexpected findings in previous hair perception research might stem from issues with the ecological validity of stimuli [25]. Cheon et al. [23] noted the lack of evidence addressing how people perceive hair and how this perception may affect romantic relationships. Consequently, developing hypotheses about the influence of hair characteristics on the perception of physical appearance is challenging. Research has concentrated on hair length and colour as the features of interest. The present research aimed to advance understanding of hair perception by examining (i) the influence of hair shine (Experiment 1) and (ii) the comparative effects of hair shine, together with systematic variation in alignment and volume (Experiment 2), on social perceptions. Although shiny hair with a smooth texture is perceived to be healthy [43], it is unclear whether these attributes influence perceptions of age and attractiveness across different hair types or colours. Accordingly, this study was exploratory, particularly concerning interaction effects between various hair features.

## METHODS

### General methodology

We conducted two experiments in which non‐expert female panellists viewed distinct expressions of specific hair features and judged them for youthfulness, health and attractiveness. Systematic stimuli were created by professional stylists using natural Caucasian hair wigs worn by the same individual. The stimuli were recorded using SOLIS (Salon Lighting Imaging System; The Procter & Gamble Company, Cincinnati, OH, USA), a custom‐built imaging system. The device is partially enclosed and provides diffuse, even lighting by incorporating six LED light bars distributed throughout the imaging space. The LEDs have a daylight‐balanced colour temperature of 5600 K with a high colour rendering index (CRI) of 95.8 and a television lighting consistency index (TLCI) of 98.2. A Canon EOS 6D Mark II digital camera (Canon Inc., Tokyo, Japan) with a Sigma 24‐105 mm lens (Sigma Corp., Kawasaki, Kanagawa, Japan) was mounted to the camera support with a gimbal. Calibration was achieved using a focus depth chart and EOS Utility software (Canon Inc., Tokyo, Japan). The imaging booth contains a stool with a centering bar, retention brackets, and positioning blocks to ensure precise orientation for participants. In Experiments 1 and 2, an online platform (AYTM; Umongous, Inc., Marlton, NJ, USA) was used to present stimulus images and record responses.

### Experiment 1

**FIGURE 1:**
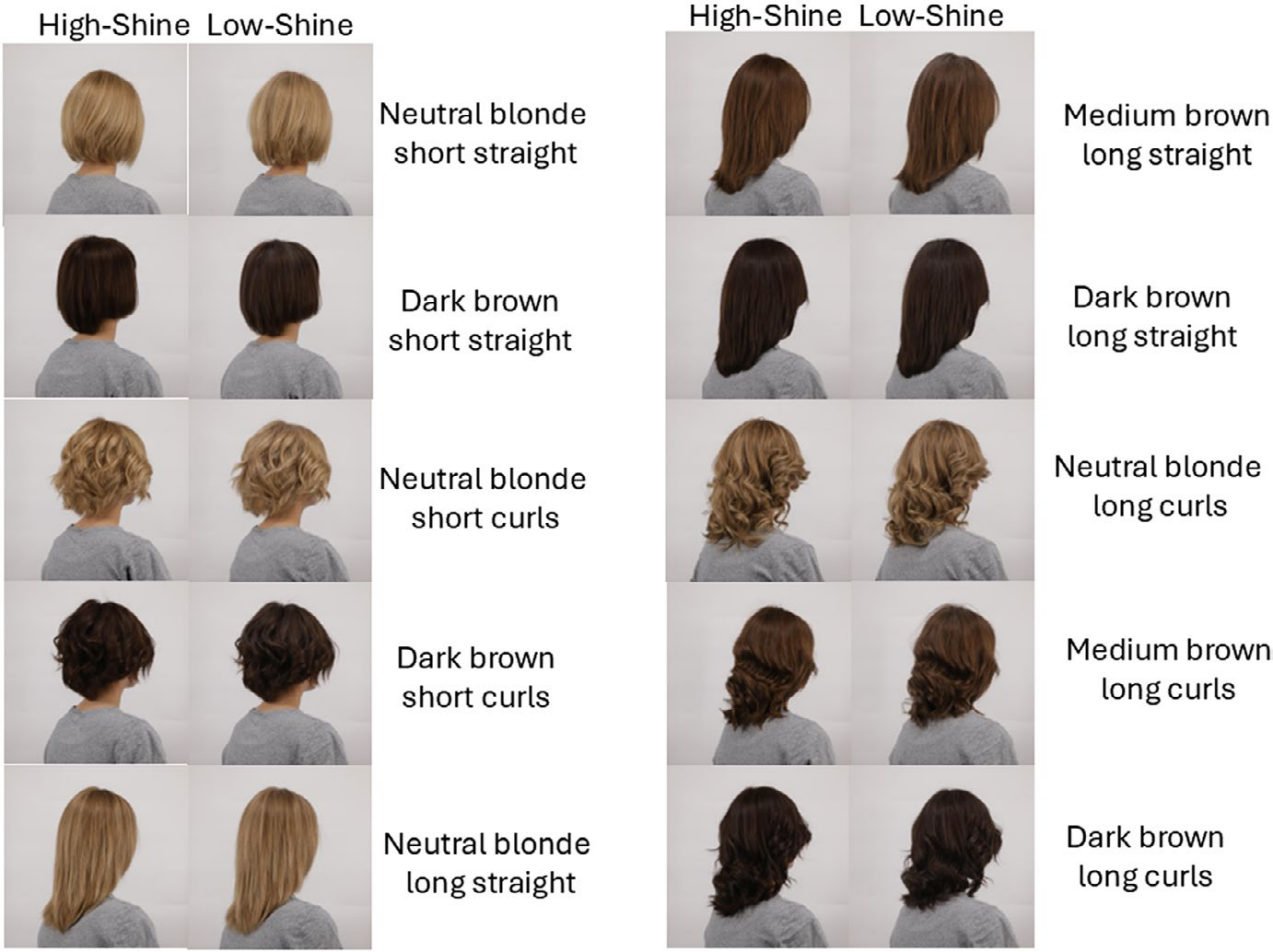
High‐ and low‐shine versions of 10 hair types, created from manipulations of natural hair wigs.

In this experiment, the research focus was on the impact of hair shine on perceptions of female physical appearance. High‐shine and low‐shine versions of wigs were created in the following conditions: neutral blonde, medium brown and dark brown hair colour; short and long hair; straight and curly hair. There was a total of 20 wigs, that is, 10 wigs each in high‐shine and low‐shine conditions (Figure 1).

The high‐shine condition of each wig was created by using a clarifying shampoo, followed by brush–blow–drying and heat straightening. For the low‐shine condition, a thin layer of dry‐shampoo spray was added evenly to the surface of the shiny hair to minimize changes to hairstyle and alignment.

A 27‐year‐old German Caucasian woman wearing each wig was photographed in a three‐quarter (3/4) back view (Figure 1) and in a neutral grey t‐shirt. Thus, facial identity was not accessible to observers. The woman was a company employee who participated voluntarily. She was debriefed about the study purposes before imaging.

Image ratings were collected from female panellists in the United States, Germany and Spain. The total sample was *n* = 1500 women (500 from each country), ranging in age from 18 to 82 years (*M* = 37.1, SD = 15.3, skew = 0.66, kurtosis = −0.49). Panellists viewed pairs of images showing a high‐shine and a low‐shine version of the same wig, with the presentation side varied. They were instructed to select the image in which the woman appeared younger, healthier or more attractive for each pair. These ratings were performed with attributes in blocks, and the order of image pairs within each block was randomized across participants. Participants were instructed at the beginning of the rating task to judge female physical appearance for several attributes. The completion time per participant was about 10 min. Participants received a reimbursement of about US$10 after completion of the rating task.

### Experiment 2

**FIGURE 2:**
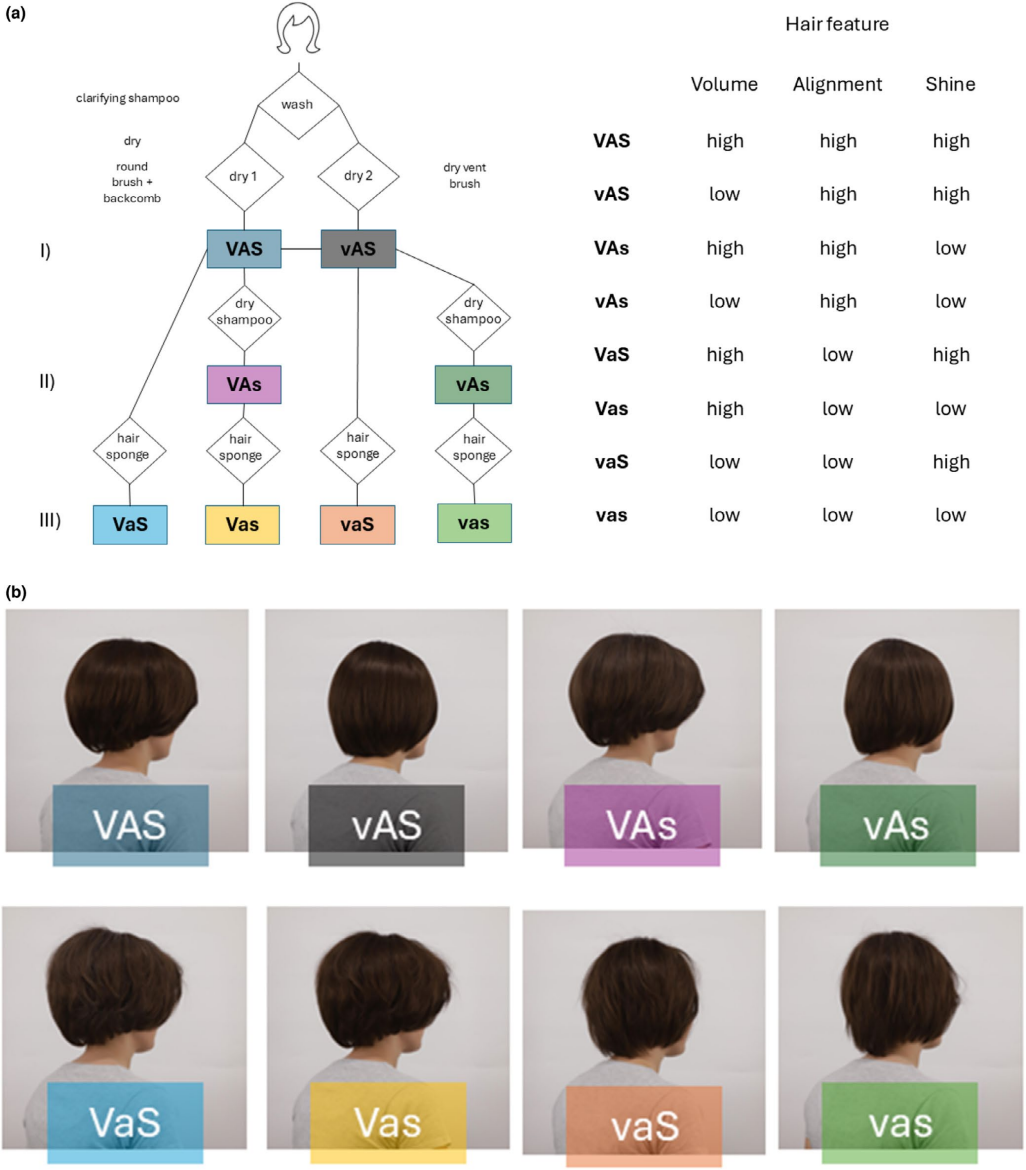
Creation process of hair feature combinations, including high/low versions of volume, alignment, and shine (a), and the female model wearing the wig in each of the eight hair feature conditions (b). Capital letter = high in feature; lowercase letter = low in feature.

In this experiment, we sought to expand on insights secured from the first experiment by systematically adding two hair features: alignment and volume. The principal study design for hair manipulation to create versions high and low in features was the same as in Experiment 1. Thus, we had wigs in high/low versions of shine, alignment and volume, rendering 8 (=2^3^) stimuli. These wigs were produced in two hair colours: neutral blonde and dark brown. Figure 2a provides a schematic of the process of hair condition creation. The wig was initially washed with a clarifying shampoo and dried (with a hair dryer at 1680–2000 W) by using a vent brush (low volume) or a round brush and backcombing (high volume), followed by hair straightening (at 185°C–200°C) with a small amount of hair oil, transferred from the hands to the hair surface, to maintain alignment. This hair was used to create further conditions, including either high or low volume; first, combinations of high volume/high alignment and low volume/high alignment, both low in shine through the application of dry shampoo (Figure 2a; I). Second, the high and low volume conditions (Figure 2a; II) and the low shine hair (Figure 2a; II) were used to create hair feature combinations, including low alignment (Figure 2a; III). Figure 2b shows the eight hair feature combinations in a three‐quarter back view.

**FIGURE 3:**
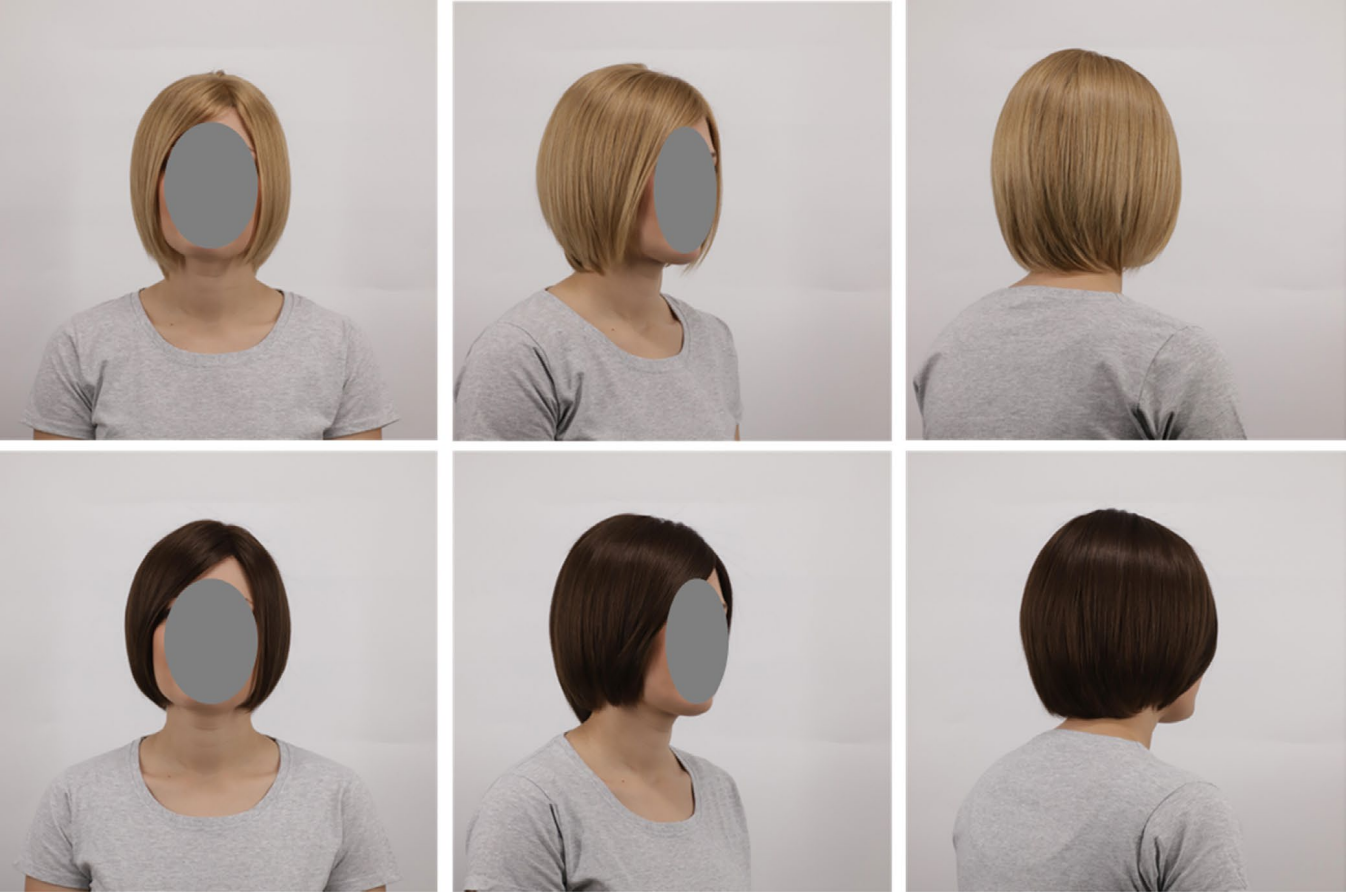
Female model, imaged from three viewpoints, wearing a neutral blonde and dark brown wig in the high shine/alignment, and low volume condition (vAS) that was rated higher for youthfulness, health and attractiveness.

A 32‐year‐old German Caucasian woman was imaged wearing these wigs in three orientations (front, 3/4 front and 3/4 back; Figure 3), always in a grey t‐shirt. The woman was a company employee who participated voluntarily. She was debriefed about the study's purposes before imaging.

Image ratings were obtained from women residing in the USA (*n* = 2000), ranging in age from 18 to 87 years (*M* = 48.4, SD = 14.9, skew = 0.06, kurtosis = −0.94). Participants viewed the images in an incomplete block design (with attributes in blocks) and judged them for youth, health, and attractiveness. Each of the 48 stimuli representing eight hair feature conditions—each in two colours and three orientations—was assessed by ~110 raters. These ratings were performed with attributes in blocks, and the order of images within each block was randomized across participants. Instructions, debriefing, and reimbursement occurred as in Experiment 1. The time to complete the ratings was 10–15 min. per participant.

## RESULTS

### Experiment 1

**FIGURE 4:**
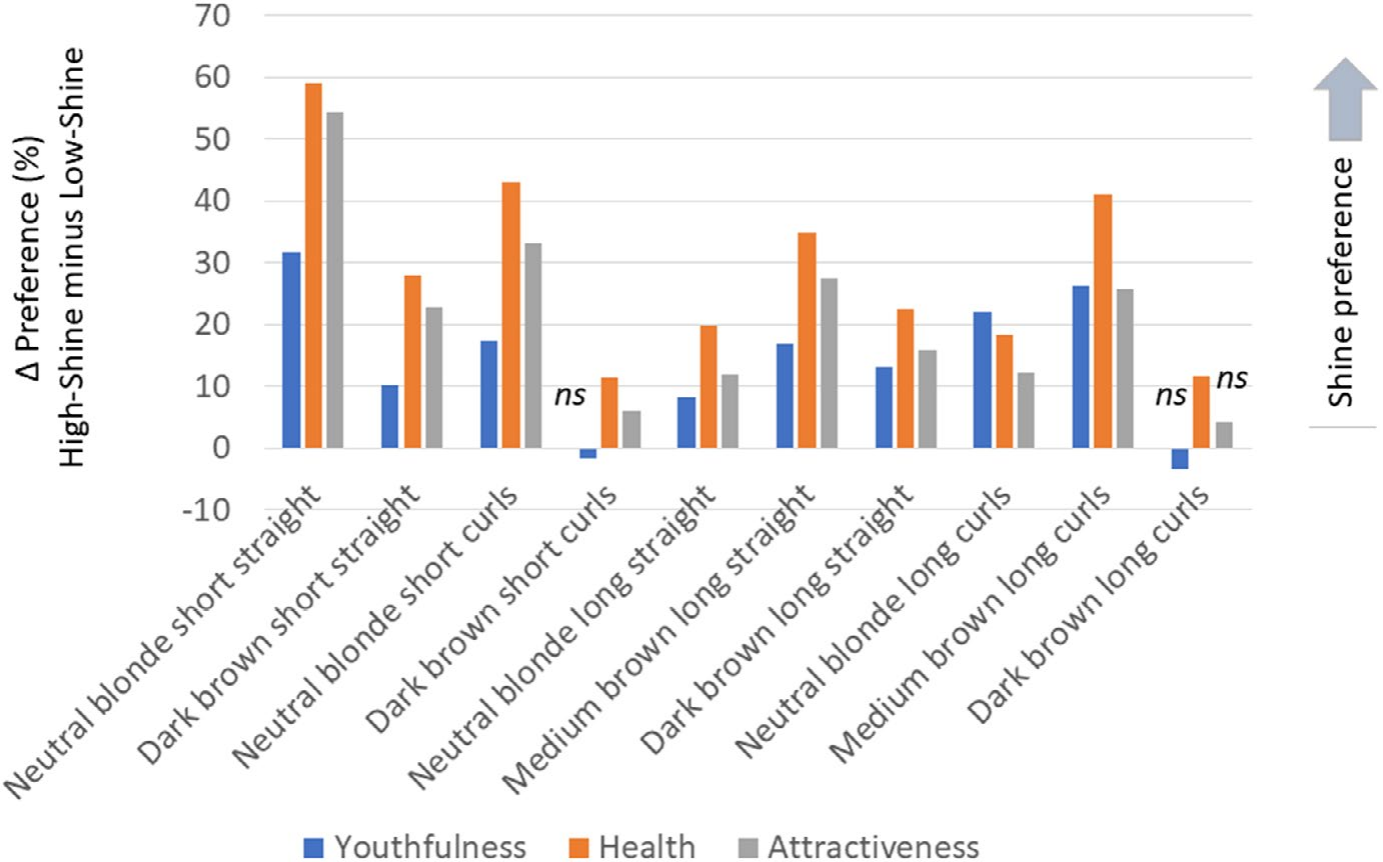
Frequency differences of youthfulness, health and attractiveness preferences for high‐shine minus low‐shine versions of hair in a sample of *n* = 1500 women from Germany, Spain and the USA.

Of the total sample (participants from the three countries), 85.6% indicated that they completed the rating study on a mobile phone and 14.0% used a PC or tablet (six participants did not provide information about the device used). We performed binomial tests with the number of choices of the high‐shine versus the low‐shine version of hair, separately for youthfulness, health and attractiveness. Considering the total sample, 8 of 10 hair images were considered more youthful in the high‐shine condition. The images of the woman in the high‐shine condition were rated healthier for all 10 hair types. Attractiveness preferences mapped onto those for youthfulness, with 8 of 10 statistically significant (high‐shine > low‐shine) (Table 1). Figure 4 visualizes the differences in % preferences for high‐shine minus low‐shine hair in the total sample.

**TABLE 1:** Counts of choices for high shine versus low shine hair in the total sample (*n* = 1500).

	High shine	Low shine	Obs. proportion	*p*
(a) Youthfulness				
Neutral blonde short straight	987	513	0.66 vs. 0.34	<0.001
Dark brown short straight	827	673	0.55 vs. 0.45	<0.001
Neutral blonde short curls	881	619	0.59 vs. 0.41	<0.001
Dark brown short curls	737	763	0.49 vs. 0.51	0.519
Neutral blonde long straight	812	688	0.54 vs. 0.46	<0.001
Medium brown long straight	876	624	0.58 vs. 0.42	<0.001
Dark brown long straight	848	652	0.57 vs. 0.43	<0.001
Neutral blonde long curls	916	584	0.61 vs. 0.39	<0.001
Medium brown long curls	946	554	0.63 vs. 0.37	<0.001
Dark brown long curls	725	775	0.48 vs. 0.52	0.206
(b) Health				
Neutral blonde short straight	1193	307	0.80 vs. 0.20	<0.001
Dark brown short straight	960	540	0.64 vs. 0.36	<0.001
Neutral blonde short curls	1073	427	0.72 vs. 0.28	<0.001
Dark brown short curls	836	664	0.56 vs. 0.44	<0.001
Neutral blonde long straight	898	602	0.60 vs. 0.40	<0.001
Medium brown long straight	1012	488	0.67 vs. 0.33	<0.001
Dark brown long straight	920	580	0.61 vs. 0.39	<0.001
Neutral blonde long curls	887	613	0.59 vs. 0.41	<0.001
Medium brown long curls	1058	442	0.71 vs. 0.29	<0.001
Dark brown long curls	837	663	0.56 vs. 0.44	<0.001
(c) Attractiveness				
Neutral blonde short straight	1158	342	0.77 vs. 0.23	<0.001
Dark brown short straight	921	579	0.61 vs. 0.39	<0.001
Neutral blonde short curls	999	501	0.67 vs. 0.33	<0.001
Dark brown short curls	795	705	0.53 vs. 0.47	<0.05
Neutral blonde long straight	839	661	0.56 vs. 0.44	<0.001
Medium brown long straight	957	543	0.64 vs. 0.36	<0.001
Dark brown long straight	869	631	0.58 vs. 0.42	<0.001
Neutral blonde long curls	842	658	0.56 vs. 0.44	<0.001
Medium brown long curls	944	556	0.63 vs. 0.37	<0.001
Dark brown long curls	782	718	0.52 vs. 0.48	0.104

To further examine women's perceptions of hair, the preferences for high‐shine and low‐shine versions of hair were summed for each attribute and then subtracted (Δ shine = high‐shine minus low‐shine). Comparing Δ shine against zero (i.e., the assumption of no differences in preferences for high‐shine and low‐shine) indicated significant differences in the total sample for youthfulness (*M* = 1.41, SEM = 0.10, *t* = 13.43, *p* < 0.001), health (*M* = 2.90, SEM = 0.11, *t* = 27.24, *p* < 0.001) and attractiveness (*M* = 2.14, SD = 0.10, *t* = 20.80, *p* < 0.001). Conducting this analysis by country yielded similar results. In addition, multivariate tests comparing perceptions of all high‐shine and low‐shine versions and considering country as a factor indicated an effect of preference (Pillai's Trace *F* = 180.2, *p* < 0.001, $\eta_{p}^{2}$ = 0.11) but no interaction of preference × country (Pillai's Trace *F* = 0.89, *p* = 0.41, $\eta_{p}^{2}$ = 0.001). Univariate tests, performed separately for youthfulness, health and attractiveness, confirmed these findings. Thus, the preference for high‐shine over low‐shine hair extended to observations in the three countries.

### Experiment 2

The initial recording of participants' ratings in AYTM ranked the hair feature from 1 (strongest preference) to 8 (weakest preference) by attribute, hair colour and orientation. Tests for the assumption of deviation from normality (one‐sample *KS*‐test) indicated significance for the dependent variables (hair features, split by attribute). However, closer inspection of histograms and scores for skewness and kurtosis did not indicate deviations from normality (youthfulness: skew = 0.06; kurtosis = −0.97; health: skew = 0.06; kurtosis = −0.97; attractiveness: skew = 0.06; kurtosis = −0.88). Therefore, we performed an 8 (hair feature) × 2 (hair colour) × 2 (orientation) analysis of variance, separate for the three attributes, to examine main and interaction effects on participants' ratings.

Multivariate tests (with confidence intervals adjusted) indicated effects of hair feature on youthfulness (Pillai's Trace *F* = 78.33, *p* < 0.001, $\eta_{p}^{2}$ = 0.46), health (Pillai's Trace *F* = 189.36, *p* < 0.001, $\eta_{p}^{2}$ = 0.67) and attractiveness (Pillai's Trace *F* = 106.59, *p* < 0.001, $\eta_{p}^{2}$ = 0.53), suggesting differences between hair features independent of hair colour and orientation. Similar (albeit smaller) multivariate effects were detected for (two‐ and three‐way) interactions. For brevity, we report only the significance values and effect sizes. *Youthfulness*: hair feature × orientation *p* < 0.001, $\eta_{p}^{2}$ = 0.08; hair feature × hair colour *p* = 0.21, $\eta_{p}^{2}$ = 0.02; hair feature × hair colour × orientation *p* < 0.001, $\eta_{p}^{2}$ = 0.06. *Health*: hair feature × orientation *p* < 0.001, $\eta_{p}^{2}$ = 0.18; hair feature × hair colour *p* < 0.001, $\eta_{p}^{2}$ = 0.06; hair feature × hair colour × orientation *p* < 0.001, $\eta_{p}^{2}$ = 0.07. *Attractiveness*: hair feature × orientation *p* < 0.001, $\eta_{p}^{2}$ = 0.19; hair feature × hair colour *p* < 0.001, $\eta_{p}^{2}$ = 0.05; hair feature × hair colour × orientation *p* < 0.001, $\eta_{p}^{2}$ = 0.12. Collectively, these findings suggest large differences in women's assessments of youthfulness, health and attractiveness of female hair attributable to variation in volume, alignment and shine. The interactions of these hair features with hair colour and orientation are notable; however, considering effect sizes, their impact on perception is small and possibly driven by the contribution of hair feature differences.

Table 2 reports univariate tests of within‐subject effects (main and interaction terms). Mauchly's test indicated that the assumption of sphericity had been violated for the three attributes. Therefore, we report Greenhouse–Geisser corrected degrees of freedom. Post‐hoc pairwise comparison of mean differences (Tukey's test) between hair features (volume, alignment, shine) indicated omnibus significances for youthfulness, health and attractiveness. The (non‐parametric) Durbin test confirmed these differences, yielding similar results.

**TABLE 2:** Univariate within‐subject effects on perception of female hair in Experiment 2 (*n* = 2000).

	df1, df2	*F*	*p*	$\eta_{p}^{2}$
(a) Youthfulness				
Hair feature	4.8, 3187.3	155.7	<0.001	0.19
Hair feature × Hair colour	4.8, 3187.3	1.5	0.179	0.002
Hair feature × Orientation	9.7, 3187.3	12.2	<0.001	0.04
Hair feature × Hair colour × Orientation	9.7, 3187.3	4.3	<0.001	0.01
(b) Health				
Hair feature	4.9, 3211.9	296.5	<0.001	0.31
Hair feature × Hair colour	4.9, 3211.9	4.9	<0.001	0.007
Hair feature × Orientation	9.7, 3211.9	19.9	<0.001	0.06
Hair feature × Hair colour × Orientation	9.7, 3211.9	6.6	<0.001	0.02
(c) Attractiveness				
Hair feature	4.3, 2856.1	177.9	<0.001	0.21
Hair feature × Hair colour	4.3, 2856.1	2.8	<0.05	0.004
Hair feature × Orientation	8.6, 2856.1	24.0	<0.001	0.07
Hair feature × Hair colour × Orientation	8.6, 2856.1	11.6	<0.001	0.03

**FIGURE 5:**
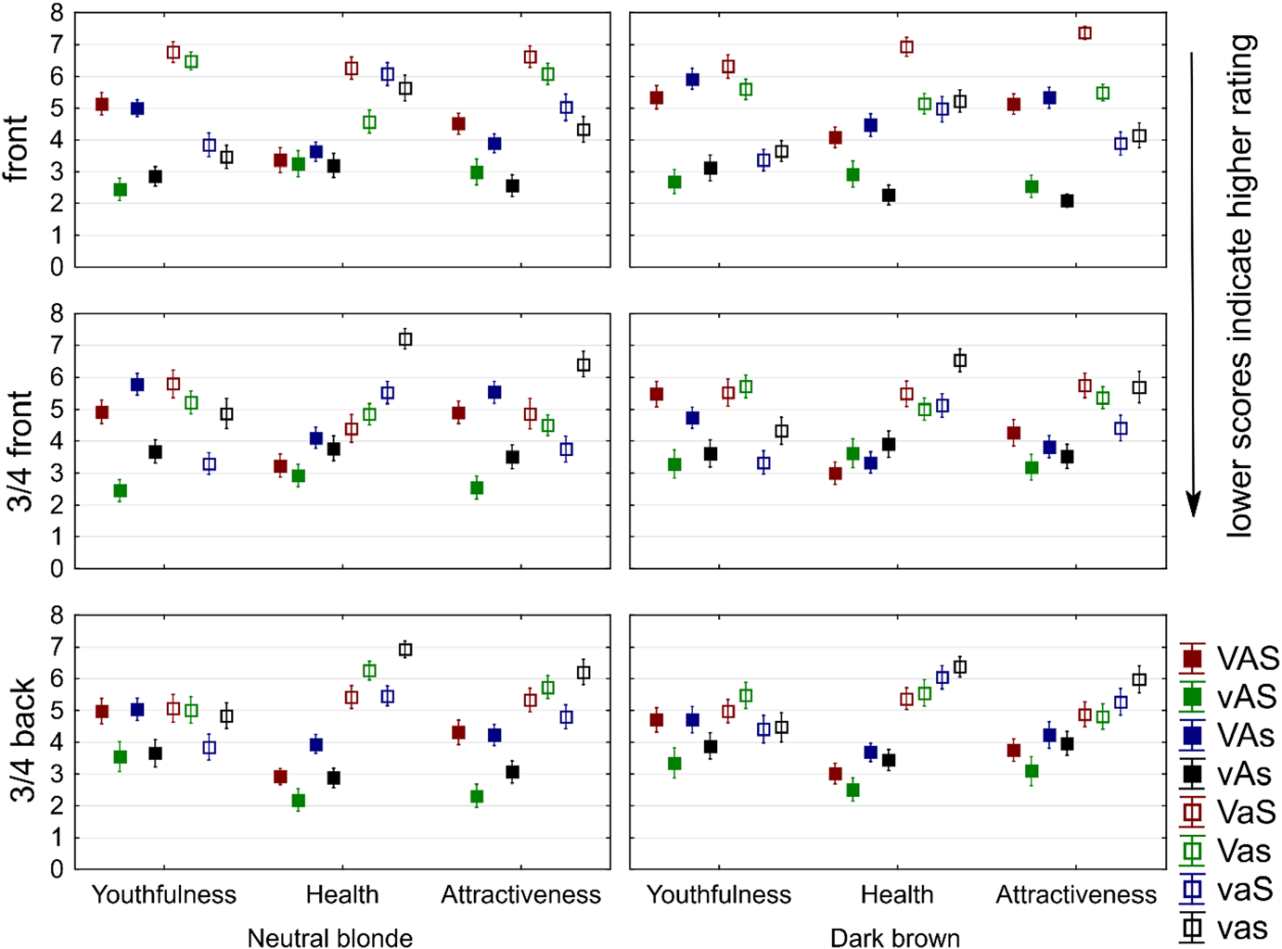
Youth, health, and attractiveness assessments (mean ranks ±2 SEM) for eight feature combinations of neutral blonde and dark brown hair in three orientations. A, hair alignment; S, hair shine; V, hair volume. Upper case letter = high in feature, lower case letter = low in feature.

Inspection of Figure 5 indicates that, with few exceptions, straight‐aligned hair in high‐shine and low‐volume condition (vAS) scores the lowest across hair colours and orientations. Additionally, straight‐aligned hair with low shine and low volume is perceived to be high on all attributes, regardless of hair colour or orientation. These findings indicate diverse effect sizes of hair characteristics on perception and underscore the minor role of hair colour and orientation, as revealed by multivariate and univariate analyses. Combinations of hair features that include high volume were evaluated less favourably.

Several patterns are noteworthy. First, (high) hair volume negatively impacted straight‐aligned and shiny hair (see vAS and vAs compared to VAS and VAs). This effect is greater for perceptions of youthfulness and attractiveness than for health. Second, feature combinations including low hair alignment (VaS, Vas, vaS, vas) are perceived as less healthy and less attractive (but not less youthful) than other hair types. Third, high shine only (together with low volume and alignment; vaS) does not lead to positive health and attractiveness assessments; high‐shine hair was perceived as youthful, though. Fourth, the differences in assessments of high‐volume hair seem to depend, in part, on orientation. In the front view, high‐volume hair is perceived as less youthful, healthy, and attractive than in 3/4 (front and back) views and for both hair colours. Fifth, comparing perceptions of hair feature combinations across hair colours shows little difference for neutral blonde and dark brown hair. Some differences between hair types may be more pronounced in one colour, but the patterns of preferences for combinations of high‐ and low‐volume, alignment, and shine seem applicable to lighter and darker hair colours. Collectively, the findings from Experiment 2 suggest (i) effects of hair features (volume, alignment, and shine) on perceptions of youthfulness, health and attractiveness, with these effects larger than for hair colour or orientation and (ii) a positive role of straight‐aligned hair in perceptions of youth, health and attractiveness, particularly in combination with high shine.

## DISCUSSION

The chemical and physical behaviour of human hair and its treatment are well understood [30, 49, 50, 51, 52, 53, 54]. However, the impact of hair features on perception has not been extensively studied. In addition to colour, shape, and style [55], head hair appearance varies with fibre condition [56, 57]. Previous research on virtual (rendered) hair documents that even subtle changes to hair diameter, density, and style impact assessments of female hair in terms of youthfulness, health and attractiveness [24]. Carefully controlled, systematic variation of hair fibre conditions in vivo is difficult to achieve. In the present study, we used natural hair wigs worn by a female model and investigated the impact of hair feature changes on perception.

Experiment 1 indicated that high‐shine versions of hair were preferred over those with lower shine. Shiny hair was rated higher for youthfulness, health and attractiveness. These preferences were observed in American and European female assessors. Experiment 2 extended the investigation of the role of hair shine on perceptions by integrating shine with alignment and volume. American women judged straight‐aligned hair, together with high shine (but low volume), as particularly youthful, healthy and attractive. These findings were obtained for neutral blonde and dark brown hair, and when viewed from different viewpoints (front, 3/4 front and 3/4 back). High volume was not rated as positively as high shine and alignment. A small but statistically significant interaction between hair feature and viewpoint suggests some dependency of hair assessment on viewpoint. However, this effect was smaller than the effects of alignment, shine and volume. Similarly, we observed interaction effects on perception for hair features, colour and viewpoint; the effect sizes for these interactions were even smaller. Collectively, the findings of the present study indicate a role for hair shine in perception, especially in combination with straight‐aligned hair. Shine and straight‐aligned hair are judged as younger, healthier and more attractive than hair low in these features. The findings are principally applicable to blonde and brown hair, and when hair is viewed from different viewpoints.

The present study corroborates previous results on the role of hair shine in assessment. Fink et al. [44] treated hair tresses in a multistage combing wheel (500 cycles) to simulate the mechanical damage from daily grooming. Undamaged versions of natural and coloured hair were perceived as younger, healthier and more attractive than damaged versions. Because Fink et al. [44] did not include hair length or style, the authors concluded that subtle differences in the optical properties between damaged and undamaged hair accounted for the impact on perception. It is well known how light interacts with hair (fibres) [45], producing a complex visual pattern comprising light reflection, absorption and scattering [46, 58, 59, 60]. Healthy (undamaged) hair differs from damaged hair in cuticle cell properties that affect light transport [43, 60].

Although damage in the Fink et al. study [44] was minimal, participants were sensitive to it and judged hair with higher shine more positively. The current study employed a more ecologically valid approach to investigate the role of shine in assessments of female hair and detected a positive impact of shine on assessments. More than 80% of respondents in Experiment 1 provided their assessments on mobile phones. This suggests that the positive visual impact of shine on hair perception is noticeable also on small screens (the average mobile phone screen size is ~16 cm). No difference between high‐shine and low‐shine versions was detected for youthfulness ratings of short and long dark brown curly hair and attractiveness of long dark brown curls, whereas for health, high shine was preferred over low shine for all hair types. This could indicate that shine plays an important role in health assessments of hair, independent of colour and length.

The findings of both Fink et al. [44] and Experiment 1 of the present study focused on manipulations of a single variable (hair shine). However, in natural conditions, the impact of head hair on female appearance may be influenced by many hair characteristics [24, 61, 62, 63]. Therefore, Experiment 2 examined a more naturalistic condition by considering hair shine together with alignment and volume. In addition, omnibus combinations of high‐ and low‐feature versions of hair were considered in two colours and imaged from different viewpoints. The visibility of shine can be influenced by colour and orientation, as the reflection and absorption of light interacting with hair fibres vary across different viewpoints [58, 60, 64]. Although these effects are well documented from the study of hair fibres and swatches [52], including swatches' movements [65], little is known about the impact of hair features other than colour and style on perceptions of hair. One of the most impactful findings of Experiment 2 was the role of hair alignment in assessments of female youth, health and attractiveness. Straight‐aligned hair was rated healthier and more attractive, especially in combination with high shine. Youthfulness ratings also were higher than those of other feature combinations, but only when hair volume was low. These findings were independent of colour and orientation. Thus, the results of Experiment 2 suggest that natural Caucasian straight‐aligned, shiny (but not voluminous) hair is judged as younger, healthier and more attractive than hair without these features. Experiment 2 revealed interactions of hair features (volume, alignment and shine) with colour and orientation; however, considering the very small effect sizes of these interactions on hair perceptions casts doubt on the ecological validity of these statistically significant findings. The principal (and largest) effects were detected for the combinations of hair volume, alignment and shine.

Previous research on the role of hair in perceptions of female physical appearance has focused on the role of hair colour; specifically, the investigation of hair colour preferences as a function of relative hair colour frequencies in a population [33]. This has led to speculations about a rare‐colour advantage effect, and related speculations that ‘gentlemen prefer blondes’ [32, 55, 66, 67]. Conclusions from studies using stimulus images have been limited due to a lack of systematic hairstyles or by the absence of capturing how light interacts with hair. The findings of the present study on the significant role of shine in the assessment of female hair suggest that the consideration of the interaction of hair fibres with light, leading to individual variation in the visual appearance of human hair, is important for assessments of a woman's youthfulness, health and attractiveness. The current findings also suggest that grooming and maintenance of hair that preserves alignment and shine lead to higher ratings of physical appearance [43, 68, 69].

The current study addresses women's perception of female hair. We instructed participants to judge female appearance for key attributes of social perception, that is, age, health and attractiveness. From an evolutionary perspective, head hair may have signalling value. Zahavi and Zahavi [70] suggest that well‐maintained hair testifies to the amount of time its owner can afford to spend caring for it. That is, individuals who are in a position, physically, financially or timewise, to allocate effort to head hair, thereby signal their quality as a mating partner and social status. Etcoff [29] contends that head hair is particularly useful in mate attraction. The author presents historical and social examples suggesting that hair is a living record of our bodies over time [71, 72, 73, 74, 75, 76]. Thus, age‐ and health‐related hair changes are informative about mating‐related quality, in addition to providing information about attitudes, preferences and personality. Taken together, hair may have a function in human mate attraction. A recent study [23] examined the functional role of hair in romantic relationships. The study showed that women with long and high‐quality hair experienced more frequent sexual intercourse with their spouses. Hair quality was rated using a single item (from ‘very bad’ to ‘very good’). The findings of this present study extend ratings of hair quality to specific features indicative of youth, health and attractiveness.

Our study has limitations that suggest the need for further investigation. The natural hair wigs used in the two experiments were European (‘Caucasian’) hair [77]. We observed similarities in assessments of female raters from three different countries. However, it is unclear whether, for hair from other ethnic groups (with different fibre characteristics [56, 78, 79]), alignment and shine play equally important roles as observed in this study. Therefore, the investigation of hair from other ethnic groups is needed to draw more definitive conclusions about the generalizability of our findings. Another limitation regards the stimuli; specifically, we used two hair feature expressions—a ‘high’ and a ‘low’ version. These versions were designed to reflect subtle differences between the two versions. From a psychometric perspective, we do not know whether the signalling strength between high and low versions of hair features is comparable. That is, a subtle difference in shine, for example, may have a stronger impact on perception than a similarly sized difference in volume. Validating feature variation on a scale is an avenue for future research on hair perception.

A word of caution is warranted regarding potential interaction effects of hair features on perceptions. The current study examined the relative impact of hair volume, alignment and shine on perceptions by creating omnibus combinations of high/low feature versions, with the general finding that high alignment (together with shine) is judged as youngest, healthiest and most attractive. Specifically, the creation of highly aligned hair is thought to affect light reflection patterns, leading to greater specular (and less diffuse) reflection that is perceived as glossy and vibrant [80]. Technical measurements of hair shine typically consider the property of the hair fibre but not the whole head [46, 52, 81]. Therefore, conclusions from the understanding of hair fibre measurement about the consumer perception of hair have remained challenging. Among other properties, hair alignment may be a key component of shine as it provides a flatter and uniform hair array for light reflection compared to less aligned (‘frizzy’) hair [82]. Heat‐straightened hair, as used in the present study for the creation of high alignment conditions (Figure 2; II), makes hair look shinier. The high degree of hair fibre alignment may override possible damage of hair fibres from heat and eventually increase shine rather than diminish it [80]. The possible combined effects of high alignment and high shine highlight the need for more research on how these factors influence perceptions of hair, given their importance to consumers who want attractive hair.

Among the strengths of the present study is the use of systematic manipulation of natural hair and the use of the same female model wearing different wigs. This removes the influence of facial information. However, future research should extend the investigation into the role of hair in assessments of female physical appearance to include women varying in skin pigmentation [83], given previous reports on interaction effects of hair and skin colour [41]. The consideration of women from different ethnic groups is also important for reasons that include ethnic variation in hair types [56, 84, 85] and the popularity of hair styles [43, 86, 87]. For example, straight hair styles are popular in Asian countries [88], which, together with the heavy pigmentation of hair, could reinforce the role of shine in its visual appearance [59, 60, 80]. In addition to considering different hair types and styles, future research on hair perception should consider the role of curls. Experiment 1 of the current study, focusing on shine, indicated differences in perceptions also for curly hair (high shine > low shine). Although the light reflection pattern of curly hair is arguably more complex than that of straight‐aligned hair, high alignment within curls may contribute to greater specular reflection and thus shine [80].

In conclusion, the current study suggests that hair alignment and shine together play a role in female assessments of youth, health and attractiveness of Caucasian women. High shine and straight‐aligned hair were perceived as more youthful and healthier. These systematic preferences were observed in three Western societies and apply to blonde and brown hair, largely independent of viewpoint and even when stimuli are rated on small screens.

## CONFLICT OF INTEREST STATEMENT

SW, MB, KK, JK, YLL, SS are employees of The Procter & Gamble Company. PJM is a retired former employee of The Procter & Gamble Company. TKS declares no conflict of interest. BF is an consultant of The Procter & Gamble Company.

## Data Availability

Research data are not shared.
